# β-Carotene: A Natural Compound Improves Cognitive Impairment and Oxidative Stress in a Mouse Model of Streptozotocin-Induced Alzheimer’s Disease

**DOI:** 10.3390/biom9090441

**Published:** 2019-09-02

**Authors:** Sundas Hira, Uzma Saleem, Fareeha Anwar, Muhammad Farhan Sohail, Zohaib Raza, Bashir Ahmad

**Affiliations:** 1 Riphah institute of Pharmaceutical Sciences, Riphah International University, Lahore Campus, Lahore 54000, Pakistan; 2 Faculty of Pharmaceutical Sciences, GC University, Faisalabad 38000, Pakistan

**Keywords:** oxidative stress, β-carotene, dementia, acetylcholinesterase, biochemical markers

## Abstract

Alzheimer’s disease (AD) is a neurodegenerative disease characterized by a cascade of changes in cognitive, behavioral, and social activities. Several areas of the brain are involved in the regulation of memory. Of most importance are the amygdala and hippocampus. Antioxidant therapy is used for the palliative treatment of different degenerative diseases like diabetes, cirrhosis, and Parkinson’s, etc. The objective of this study was to assess the effectiveness of exogenous antioxidants, in particular, β carotene (1.02 and 2.05 mg/kg) against intracerebroventricular injected streptozotocin-induced memory impairment in mice. Streptozotocin (3 mg/kg, i.c.v) was administered in two separate doses (on 1st and 3rd days of treatment) for neurodegeneration. Fifty Albino mice (male) were selected in the protocol, and they were classified into five groups (Group I—control, Group II—disease, Group III—standard, Group IV–V—β-carotene-treated) to investigate the cognitive enhancement effect of selected antioxidants. The cognitive performance was observed following the elevated plus-maze, passive avoidance, and open field paradigms. Acetylcholine esterase, β-amyloid protein, and biochemical markers of oxidative stress such as glutathione peroxidase, superoxide dismutase, and catalase were analyzed in brain homogenates. In silico activity against acetylcholinesterase (AChE) was determined by the molecular modeling of β-carotene. β-carotene at a dose of 2.05 mg/kg was found to attenuate the deleterious effects of streptozotocin-induced behavioral and biochemical impairments, including the inhibition of acetylcholinesterase activity. The in silico studies confirmed the binding capacity of β-carotene with the acetylcholinesterase enzyme. The administration of β-carotene attenuated streptozotocin-induced cognitive deficit via its anti-oxidative effects, inhibition of acetylcholinesterase, and the reduction of amyloid β-protein fragments. These results suggest that β-carotene could be useful for the treatment of neurodegenerative diseases such as Alzheimer’s disease.

## 1. Introduction

Alzheimer’s disease (AD) is a neurodegenerative disease characterized by a cascade of changes in cognitive, behavioral, and social activities [1]. Numerous neurotransmitters and neuromodulators imparting their role in cognition are acetylcholine, serotonin, GABA (γ-aminobutyric acid), estrogen, neuropeptides, nitric oxide, endogenous antioxidants, and calcium [2]. Cholinergic transmission is involved in the severity of cognitive abilities [3]. Dwindled cholinergic function leads to cognitive impairments. Various anticholinesterase drugs are reported to alleviate AD by enhancing the acetylcholine concentration in the brain [4]. Acetylcholine (ACh) regulates many functions in the Central nervous system, such as alertness, wakefulness, and aggression [5]. ACh exerts its effect on the striatum, hippocampus, and amygdala [6]. The main regions of the brain involved in memory and learning processes are the basal forebrain and hippocampus [7]. Any distortion in the function or structure of these brain areas could cause cognitive impairment. The amygdala and hippocampus interact to regulate the memory system when emotions are involved [8]. These distortions lead to disruptions in the amyloid precursor protein (APP) metabolism and β-amyloid production that causes neurotoxicity [9]. Recent research indicated that the major contributing proteins in Alzheimer’s are tau, β-amyloid, and neurofibrillary tangles [10]. Any deformities in APP and amyloid production or destruction lead the AD. Different therapies in clinical trials for the treatment of AD are anti-amyloid and anti-tau therapies [11]. Streptozotocin is a natural antibiotic and is toxic to cells. Intracerebroventricular administration of streptozotocin results in a cognitive deficit. It causes the aggregation of Aβ and tau proteins and diminished cholinergic activity that is responsible for a cascade of pathological reactions leading to dementia. This cognitive decline may be associated with the reduced concentration of glycogen synthase kinase.

The free radical generation and oxidative damage theories suggest that oxidative stress promotes neurodegeneration that is related to Alzheimer’s [12]. Oxidative damage occurs due to the disturbance between reactive oxygen species (ROS) generation and the body’s ability to remove these ROS, as ROS could lead to tissue damage [13,14]. The brain areas such as the cerebral cortex and hippocampus that regulate cognitive and motor function are more vulnerable to oxidative stress and require antioxidants [15]. Another possible mechanism of neurodegeneration may be the inflammation of neurons, increased reactive oxygen species leading to oxidative damage, and biochemical modifications in the brain that ultimately lead to neuronal death [16].

Antioxidants could be exogenous or endogenous and perform various functions [17]. Antioxidants perform their function by acting as precursors, scavengers, or by removing oxygen species. They impart a role by inhibiting the synthesis of reactive oxygen species and by forming complexes with metal ions that are involved for the catalysis of reactive oxygen species generation [18]. There are two kinds of antioxidant systems present naturally in the body, i.e., various enzymes and antioxidants with low molecular weight (LMWA). The enzymes which act as antioxidants are glutathione, peroxidase, catalase, and superoxide dismutase [19]. The low molecular weight antioxidants (LMWA) are of two type’s, i.e., direct and indirect-acting. Direct-acting LMWA are scavengers and chain-splitting antioxidants [20].

β-carotene belongs to the class of carotenoid hydrocarbons. β-carotene acts as an antioxidant due to its scavenger and quencher nature, as well as its synergistic effect with other antioxidants [21]. The current study was carried out to evaluate the effectiveness of β-carotene on cognitive performance by using behavioral paradigms and endogenous antioxidant markers because carotenoids possess t antioxidant effects which armor against lipid peroxidation and oxidative stress.

## 2. Materials and Methods

### 2.1. Materials

β-Carotene, trichloroacetic acid solution, 5,5-dithio-bis-(2-nitrobenzoic acid) (DTNB) reagent, phosphate buffer solution, pyrogallol solution, dipotassium hydrogen phosphate, potassium hydroxide, potassium dihydrogen phosphate, hydrogen peroxide from Sigma Aldrich, Darmstadt, Germany and diazepam from Pfizer, Karachi, Pakistan. All chemicals were purchased from the local market.

### 2.2. Experimental Animals

Male mice (n = 50) were received from the Veterinary Research Institute (VRI), Lahore. All mice were almost two to three months old weighing 30–40 g at the start of the study. All animals were kept at Riphah International University for two weeks prior to the study under light/dark cycle, 45–55% humidity, and at a temperature of 22 ± 2 °C for acclimatization. They were provided with water and food ad libitum. This study was carried out on animals after obtaining permission from the research ethical committee (REC) of Riphah International University with an authorized number of REC/RIPSLHR/2017/012 ruled under the regulation of the Institute of Laboratory Animal Resources, Commission on Life Sciences University, National Research Council (1996).

### 2.3. Study Design

They were categorized into five groups each containing 10 mice. Group I, II, and III served as the control group, disease group (streptozotocin 3 mg/kg i.c.v—intracerebroventricular injection), and standard (piracetam 200 mg/kg. i.p.—intraperitoneally) groups, respectively. Group IV & V served as treatment groups (β-carotene at a dose of 1.02 and 2.05 mg/kg, respectively).The dose of β-carotene was selected by considering the available human dose that is converted to the animal dose [22]. β-carotene was administered in the form of a suspension in CMC (Carboxy methyl cellulose). Streptozotocin (STZ) was administered i.c.v bilaterally in two divided doses (1st and 3rd day of treatment). The groups were given the treatment orally for fourteen consecutive days. On day 14, each animal was assessed for cognitive function.

### 2.4. Evaluation of Cognitive Performance

#### 2.4.1. Elevated Plus-Maze

The apparatus used for the elevated plus-maze was made up of four arms, i.e., two open arms (16 × 5 cm) and two closed arms (16 × 5 × 12 cm) that were extended from the central stage (5 × 5 cm) with a height of 25 cm. On the fourteenth day, after one hour of administration of the oral dose, the animals were tested by placing each mouse at edge of open arm, facing away from central stage and the transfer latency was measured for 90 s. The transfer latency is the time (s) the mice took to enter from the open arm into the closed arm. The retention of memory was measured by performing the same procedure on the 15th day (after 24 h) [23].

#### 2.4.2. Passive Avoidance Apparatus

The passive avoidance apparatus is made up of a box (27 × 27 × 27 cm). The box has four walls.

One wall is made up of Plexiglas and three wooden walls. A platform of wood (10 × 7 × 1.7 cm) is placed in middle of the floor. The floor is made up of 3 mm SS—stainless steel rod each with a distance of 8 mm. A 20 volts AC electric shock was applied to the floor. On the fourteenth day after one hour of administration of the oral dose, each experimental mouse was treated for their cognitive performance. The test was divided into three sessions (first, second, and retention sessions). In the first session, each experimental animal was placed onto the wooden platform and the step down latency was measured. The step down latency (SDL) is the time the animal took to step down with four paws from the platform to the floor. The experimental animal that had a SDL within 2–15 s was taken for the second and retention tests. After 90 min of the first test, another session was carried out. The animals with a SDL of more than 60 s were selected for the retention test. The retention test was performed after 24 h on the 15th day without electric shocks. The SDL was noted in the same manner as previously recorded. The cut-off time was 300 s. An increased SDL was an indication of cognitive enhancement [24].

#### 2.4.3. Open Field Apparatus

The open field apparatus is composed of a square field (60 × 60) with a white floor which is segregated into 36 squares (10 × 10) enclosed by a black Plexiglass wall (25 cm high). A red light is positioned over its center to illuminate the field. The 20 squares which are adjacent to the wall are named as the “field periphery”. The field periphery represents a protected field and the remaining 16 squares represent an “exposed field” or field center.

On day 14, after one hour of administration of the oral dose, test was conducted during the first half of the dark phase of the light/dark cycle. Each mouse was placed in the center allowing it to move freely across the entire field for 5 min [25].

To examine the cognitive performance, different parameters of the open field were classified as follows;

##### Behavior

A. Whole or major body movement

1. Type of movement

- Latency (Initial time taken by the mice to leave the start area)
- Freezing (Time spent by the mice without any movement)
- Rearing

2. Part body Movement

a. Scratching/Grooming

##### Location

- Field area visited (central and peripheral)
- Crossing

##### Autonomic Nervous System (ANS)

- Urination
- Defecation [26].

### 2.5. Neurochemical Studies

#### 2.5.1. Estimation of Reduced Glutathione (GSH)

A total of 1 mL of brain homogenate was taken and it was precipitated with 1 mL of 10% trichloroacetic acid (TCA). Then, 0.5 mL of DTNB reagent (0.1 mM) and 4 mL of phosphate solution were mixed with an aliquot of supernatant. The sample mixture was examined under a UV spectrophotometer at 412 nm.

The following Equation was used for the measurement of glutathione peroxidase:

GSH = Y − 0.00314/0.034 × DF/BT × VU
(1)

In the above equation, DF denotes dilution factor that is 1, VU represents aliquot volume, Y is the absorbance, and BT = Brain tissue homogenate [27].

#### 2.5.2. Estimation of Oxidized Glutathione (GSSG)

A total of 1 mL of tissue homogenate was mixed with reaction buffer consisting of 0.1 mM of DTNB, 0.5 mM of NADPH, and 2 mM of GSSG. The rate of DTNB reduction was monitored for 5 min at 25 °C by a spectrophotometric method at 412 nm. The blank contained all except GSSG [28]. The standard curve was used for the estimation of GSSG levels:

Y = 0.0174X + 0.0023
(2)

GSSG/GSH redox ratios were determined by dividing the GSSG values by the GSH levels [29].

#### 2.5.3. Determination of Superoxide Dismutase (SOD)

A reaction mixture (3 mL) was made by adding 0.1 mL of tissue homogenate, 0.1 mL of pyrogallol solution (1 M), and 2.8 mL of potassium phosphate buffer (pH 7.4 and 0.1 M). The sample was analyzed at a wavelength of 312 nm [30].

The regression equation of SOD is as follows:

Y = 0.0095x + 0.1939(3)

#### 2.5.4. Estimation of CAT

A total of 1.95 mL of phosphate buffer (pH 7, 50 mM) and 1 mL H_2_O_2_ solution (30 mM) were mixed with 0.05 mL of tissue homogenate to note the absorbance of Catalase which was estimated by using the following formula:

(4) CAT=δO.D÷E×Volume of sample(mL)×protein(mg)

δO.D represents the change in absorbance per minute, E symbolizes the extinction coefficient of H_2_O_2_ with a value 0.071 mmol cm^−1^ [31]. The Lowery method was followed to measure the protein contents [32]. The regression line followed to estimate the contents of protein was as follows:

Y = 0.00007571x + 0.0000476(5)

#### 2.5.5. Determination of Acetylcholinesterase Activity

A total of 2.6 ml phosphate buffer (pH = 8) and 100 µL of DTNB reagent (0.1 mM) was added to 0.4 mL of tissue homogenate. The absorbance was noted at 412 nm. The initial reading was noted when the absorbance of the constituents stabilized. Then, 20 µL of acetylthiocholineiodide (1 mM) (substrate) was mixed with the contents of the cuvette and the absorbance was noted for 10 min every 20 min. The mean change in absorbance was calculated by using the following formula:

R = 5.74 × 10^−4^ × A/Co (6)

A = change in absorbance per minute.

Rate of moles of acetylthiocholiniodide hydrolyzed per min/gm of brain tissue.

C_o_ = Original concentration [33].

### 2.6. Protein Analysis by ELISA

An ELISA kit was used for the determination of Aβ1-40 and Aβ1-42 levels in the brain tissues of mice. Aβ1-40 and Aβ1-42 were combined with HRP—horseradish peroxidase enzyme to make the antigen-antibody-enzyme-antibody complex. After that, TBM (3,3′,5,5′-tetramethylbenzidine) solution was added. This reaction was stopped by the addition of sulfuric acid. The change in color was observed at 450 nm spectrophotometrically. The concentrations of Aβ1-40 and Aβ1-42 were quantified by comparing the optical densities of the samples to the standard [34].

### 2.7. In Silico Modeling

β-carotene was computationally modeled to predict its modulatory behavior on acetylcholinesterase (AChE) via docking in Autodock Vina 1.1.2 [35]. The three dimensional (3D) conformers of β-carotene (CID: 5280489) and Piracetam (CID: 4843) were retrieved from Pubchem database and saved as PDB—protein data bank formats using Open Babel Package 2.3.1 [36]. The crystallized structure of AChE (PDB ID: 2C5G, 1.95 Å) was retrieved from the (RSCB = Research Collaboratory for Structural Bioinformatics) protein data bank (https://www.rcsb.org/). The high-energy atomic conformational strains were minimized by CHARMm (Chemistry at HARvard Macromolecular Mechanics )forcefield with the MMFF94 partial charge method to optimize the structures of ligands and AChE in Accelrys discovery studio visualizer v17.2 [37]. The rigid protein was prepared for flexible ligands in Autodock tools 1.5.6. The polar hydrogens were added, and non-polar hydrogen was merged to optimize the tautomeric and ionization states of protein residues. The search space was defined by 40 * 40 * 40 (x,y,z) dimensions of a grid box volume with 1 Å grid point spacing and centered to 4.61932 * 65.9682 * 55.4541 (x,y,z) coordinates. The binding energy (ΔG) of the ligand to protein was estimated by the scoring function of the Autodock Vina 1.1.2. The accuracy of the docking protocol to predict the global conformational minimum was validated by re-docking of crystallized ligand (i.e., thiocholine) of 2C5G and the comparison with its apo conformation. Docking was performed in triplicates for each ligand and binding posed with the lowest binding energy was considered to calculate the inhibition constant (Ki) (5). These binding conformations were further utilized to analyze the interactions of the ligand-protein complex using Accelrys discovery studio visualizer v17.2.

### 2.8. Statistical Analysis

All the results were represented as mean ± SEM (n = 10) by using the Graph Pad Prism software version 5, San Diego, CA, USA. The parametric test was applied to the animal behavioral analysis. Data described in Table 1 and Table 2 were analyzed by applying the one way ANOVA. *p* ≤ 0.05, *p* ≤ 0.01, and *p* ≤ 0.001 were considered as significant (*), moderately significant (**), and highly significant (***), respectively, compared to the disease group.

## 3. Results

### 3.1. Evaluation of Cognitive Performance

#### 3.1.1. The Effect of β-Carotene on the Transfer Latency Using the Elevated Plus Maze Model in Mice that Received i.c.v STZ

**Figure 1:**
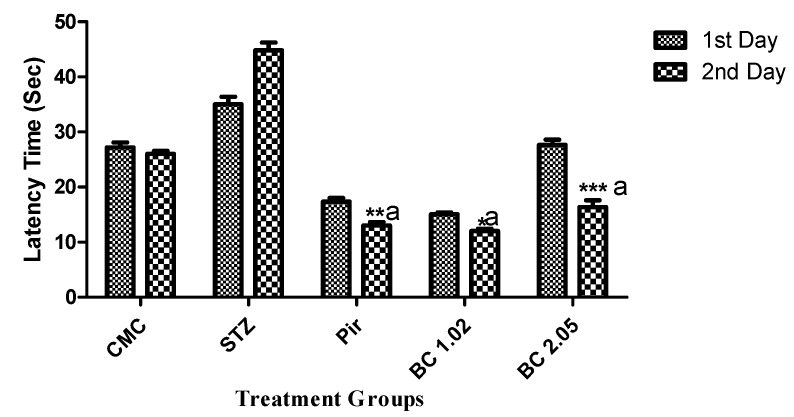
Effect of β-carotene on the transfer latency using the elevated plus maze model in mice that received i.c.v STZ—streptozotocin. Data are represented as mean ± SEM, n = 10, ^a^ *P* < 0.05. Significance was given in comparison to the disease group and *p* < 0.05 *, 0.01 **, 0.001 *** was given when compared with day one. STZ = Streptozotocin, Pir = Piracetam, BC = β-carotene.

Cognitive performance was assessed by following the elevated plus maze paradigm. The animals were subjected to transfer latency evaluation on the 15th day. Figure 1 clearly indicated that animals that received β-carotene (1.02 and 2.05 mg/kg) caused highly significant (*p* ≤ 0.001) variation of transfer latency (TL) in mice compared to the diseased control group.

#### 3.1.2. Effect of β-Carotene on the Step Down Latency Using the Passive Avoidance Model in Mice that Received i.c.v STZ

**Figure 2:**
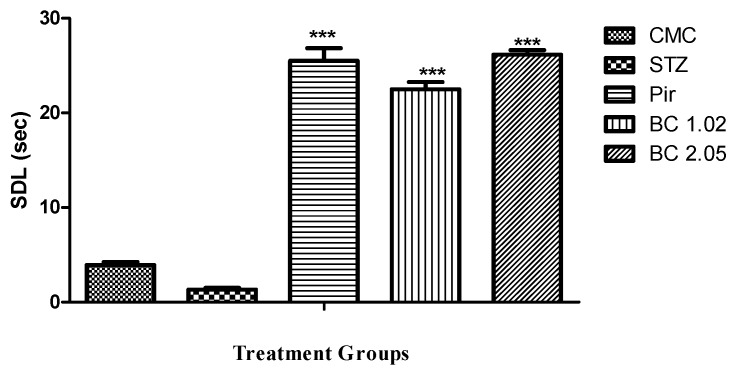
Effect of β-carotene on the step down latency using the passive avoidance model in mice that received i.c.v STZ. Data are represented as mean ± SEM, n = 10. Significance was given in comparison to the disease group. *** *P* < 0.001 was given in comparison to disease group.

The passive avoidance model has been used to examine the long-term memory based on the step down latency. It was clear from Figure 2 that the mice treated with β-carotene (1.02 and 2.02 mg/kg) showed moderately significant (*p* ≤ 0.01) improvement in cognitive performance.

#### 3.1.3. The Effect of β-Carotene Following the Open Field Paradigm in Mice that Received i.c.v STZ

Different parameters such as whole body movement, partial body movement, location, and ANS are observed with the help of the open field paradigm. Table 1 revealed that β-carotene (2.05 mg/kg) showed highly significant improvement (*p* ≤ 0.001) in freezing, central, and peripheral area visited, defecation, and urination. Low-dose β-carotene (1.02 mg/kg) showed moderately significant (*p* ≤ 0.01) improvement in rearing and highly significant improvement in all other parameters.

### 3.2. Determination of Biochemical Markers in Brain Tissues of Mice that Received I.STZ

**Figure 3:**
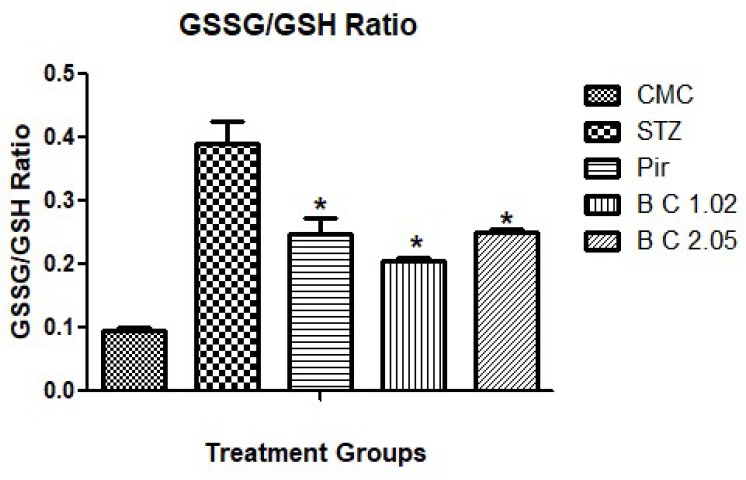
GSSG/GSH ratio of the treated groups. Data are presented as mean ± SEM, n = 10, * *p* < 0.05 as compared to the disease group (STZ).

Biochemical markers such as GSH, SOD, CAT, and acetylcholinesterase activity were determined to estimate the efficacy of selected antioxidants in oxidative stress. GSSG/GSH ratio was also estimated. Results indicated significantly increased levels of all antioxidant enzymes and decreased acetylcholinesterase activity of the treated groups as compared with the diseased control group (Table 2). GSSG/GSH ratio was significantly decreased in all treated groups when compared with diseased group (Figure 3).

### 3.3. Protein Analysis by ELISA

**Figure 4:**
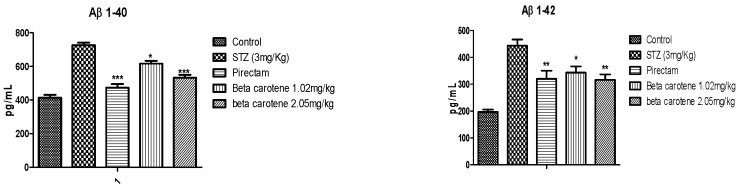
Total Aβ 1-40 and Aβ 1-42 levels extracted from the mouse brain. * *P* < 0.05, ** *P* < 0.01 and *** *P* < 0.001 Significance was given in comparison to the disease group.

β-amyloid estimation by the ELISA kit method indicated that β-carotene significantly reduced the levels in the mouse brain. This reduction might be due to less aggregation and plaque formation in the brain (Figure 4).

### 3.4. In Silico Modeling

The molecular modeling of β-carotene was performed to support its in-vitro activity against acetylcholine esterase (AChE). The re-docking of the flexible crystallized ligand validated the credibility of the results generated by the employed docking protocol. The re-docking simulation provided a binding conformation quite similar to the apo-conformation of the native ligand with 0.4129 Å of root mean square deviation (RMSD) at the active site (Figure 4). The binding conformation of the re-docking also preserved the conserved interactions of the apo conformation with PHE330, TRP84, HIS440, and GLU199 protein residues at the catalytic site.

Following the validation, β-carotene was simulated as a test ligand in the docking protocol with Piracetam as a standard. The lowest binding energy (ΔG) served as a function of higher affinity towards the protein (i.e., AChE—acetylcholine esterase). The docking simulation of Piracetam indicated the −5.8 kcal/mol of binding energy (ΔG) as a measure of its binding affinity towards AChE to inhibit its activity with 56.05 μM of inhibition constant (Ki). However, the binding affinity of β-carotene was found to exceed the standard threshold with −7.7 kcal/mol of binding energy (ΔG) and superior 2.27 μM inhibitions constant as compared to Piracetam. The key parameters of the docking simulation for these ligands have been tabulated (Table 3) that accounts for their potency towards AChE.

**Figure 5:**
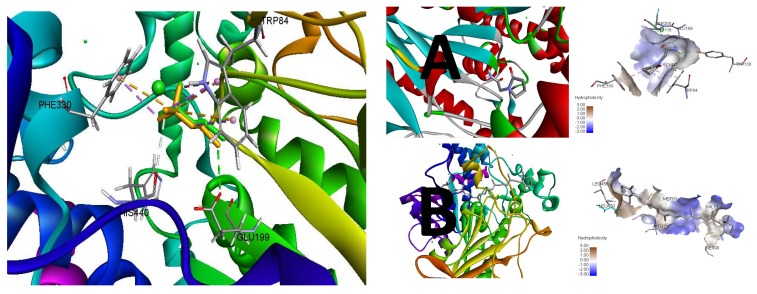
Re-docking of the flexible crystallized ligand for comparative conformational alignment with respect to the apo conformation. Best binding mode simulated (**Left**) and the pattern of hydrophobic interactions (**Right**) of Piracetam (**A**) and β-carotene (**B**) at the active site of AChE.

The binding energy of Piracetam was found to be distributed into energy components of hydrogen bonding and hydrophobic interactions at the catalytic site. On the other hand, β-carotene predominantly interacts with AChE by hydrophobic interactions that may account as a sole energy component of its binding energy (Figure 5). However, β-carotene shares the higher hydrophobic interactions with AChE as compared to Piracetam that exceeds the binding energy contribution of both energy components of Piracetam at the catalytic site.

**Figure 6:**
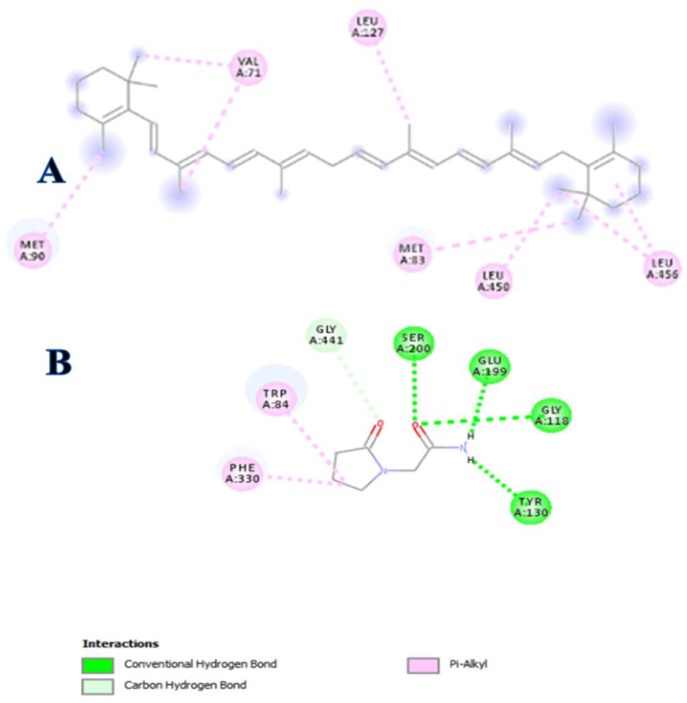
Two-dimensional (2D) plot of Piracetam (**B**) and β-carotene (**A**) binding interactions with residues of AChE active site.

The inhibitory potential of Piracetam was found to be consistent with its interaction to conserved PHE330, TRP84, and GLU199 protein residues of thiocholine at the catalytic site of AChE. In addition, its pyrrolidine and acetamide moieties were also involved in the hydrogen binding to GLY441, SER200, GLY118, and TYR130. Comparatively, β-carotene exhibited the distinct modulatory behavior by hydrophobic interactions with unique LEU456, LEU450, LEU127, MET83, VAL71, and MET90 protein residues of AChE as compared to the conserved residues of the catalytic site (Figure 6).

## 4. Discussion

Cognitive impairment is a broad term related to many neurodegenerative diseases such as Alzheimer’s. Present research work was carried out to evaluate the antioxidant potential in streptozotocin-induced memory impairment in experimental animals.

It is evident from previous studies that oxidative stress has a critical role in neurodegenerative disorders. Oxidative stress causes the release of glucocorticoids from adrenal glands, which is attributed to damage of the neurons and the impairment of memory [38]. Oxidative stress occurred due to the imbalance between ROS generation and the body’s ability to remove these ROS, as ROS can lead to tissue damage that has been a suggested mechanism for cognitive impairment due to normal aging and neurodegenerative diseases [39]. The antioxidant’s potential to improve cognition depends upon its ability to reach the brain [40].

Endogenous antioxidant levels revealed a greater increase in GSH, SOD, and CAT after treating the groups with exogenous antioxidants, such as β-carotene that had received i.c.v streptozotocin.

It is well established that the levels of GSH, CAT, and SOD were decreased in diseased animals leading to oxidative damage. It may be possible that i.c.v STZ could lead to the production of ROS, such as superoxides that are ultimately responsible for brain damage. Superoxides lead to reduced concentrations of glutathione. Glutathione (GSH) is the only defense that is available in mitochondria to metabolize hydrogen peroxide [41].

GSH reduction may be due to increased utilization, decreased formation, or increased metabolism of GSH [42]. Glutathione has a vital role in the management of oxidative stress and maintain the normal homoeostasis [43]. The GSSG/GSH ratio is a good indicator for monitoring the antioxidant intervention efficacy [29]. The same has been found in the present study as the ratio decreased significantly in Pirecetam, beta carotene 1.02 and 2.05 groups when compared with the STZ-treated animals. Administration of i.c.v STZ results in oxidative stress. Superoxide is most common among all free radicals that are generated during oxidative phosphorylation. When molecular oxygen is converted to water during normal cell metabolism, reactive oxygen species are produced (ROS). These ROS are superoxide, hydroxyl, and hydrogen peroxide radicals. Catalases degrade H_2_O_2_ into water [44].

SOD is considered to be the first-line of defense against free radicals. The increased concentration of SOD was found in β-carotene-treated mice, which may relate to the reversal of cognitive impairment caused by oxidative damage. The reduced level of SOD in diseased mice may relate the aggressiveness with oxidative damage due to stress. The cognitive enhancement effects may be attributed to the increased levels of endogenous antioxidants by reducing the oxidative stress [45].

Decreased energy metabolism or oxidative stress causes cognitive impairment by inhibition of ATP and acetyl—CoA synthesis that ultimately results in cholinergic deficiency. This cholinergic deficiency is primarily due to the increased activity of cholinesterase activity in the brain and decreased cholinecacetyl transferase activity in the hippocampus of i.c.v STZ mice [46]. Antioxidants have been the main therapeutic agents for several neurodegenerative diseases [47]. Acetylcholine owes to its excitatory effect causes the contraction of muscles and the secretion of hormones and regulates many functions in the CNS, such as alertness, wakefulness, and in aggression [5]. ACh has a vital role in improving cognitive function. The acetylcholine release is dynamic in different neural regions. ACh exerts its effect on the striatum, hippocampus, and amygdala. These three neural systems are involved in cognitive function. The release of ACh has a vital role in modulating the balance between the various neural systems during the training process, ACh releases immediately in the hippocampus earlier in the training process; however, Ach release is slower in the striatum and is involved in the extensive training [48,49]. Amyloid β protein (Aβ) aggregation and plaque formation are the principal biomarkers for the detection of Alzheimer’s disease. The most abundant form of Aβ protein in Alzheimer’s is Aβ1-40 and Aβ1-42 [50]. Among two Aβ1-42 had special importance in Alzheimer’s disease as it is early deposited as a plaque form in Alzheimer [34]. Investigation of these isoforms of β-amyloid proteins showed significant reduction in the mice brains treated with β-carotene. Following experimental studies, the computational studies were performed to explain the AChE inhibitory potential of β-carotene. The molecular docking is widely used to predict the binding affinity and simulate the ligand-target complex orientations and interactions to provide the structural insights into the mechanism of action of ligand to modulate the target activity. In this study, a reliable docking protocol was adopted to provide the insights into the mechanism of action of β-carotene to inhibit the AChE activity with reference to Piracetam as a standard. Comparatively, the β-carotene exhibited the superior binding affinity with the lowest binding energy (ΔG) and inhibition constant (Ki). The hydrophobic energy components of β-carotene oriented it with the lowest binding energy as compared to hydrogen bonding and hydrophobic energy components for Piracetam. β-carotene is hydrophobic in nature with no hydrogen bond donor and acceptor on its skeleton that may justify its binding energy solely relying on hydrophobic interactions. Moreover, this hydrophobic behavior may suggest its repulsion from active site polar components involved in hydrogen bonding and subsequent diversion from conserved active site residues to form the interactions with unique residues. Therefore, these structural insights can reasonably justify the allosteric behavior of β-carotene to modulate the activity of AChE. For new drug discovery, the popularity of in silico modeling techniques is increasing day by day. This computational approach can facilitate the researcher for finding the new drug candidates against selected targets. β-carotene is effective naturally occurring antioxidant. Due to its antioxidant property, it may have the ability to treat neurodegeneration-related disorders. This study has been evaluated for its binding capability as a ligand to acetylcholinesterase enzyme. The decreased concentration of this enzyme in β-carotene-treated mice as compared to the diseased group may relate with the anti-Alzheimer’s effect and support in silico profiling of β-carotene against the acetylcholinesterase enzyme [51,52].

## 5. Conclusions

The administration of β-carotene in STZ-induced cognitive deficit mice prevented the decrease in ACh concentration by inhibiting the acetylcholinesterase enzyme. Reduced plaques of β-amyloid protein in brains of β-carotene-treated mice also indicated the improvement of cognitive function. In silico studies also confirmed the binding capability of β-carotene with the acetylcholine esterase enzyme. Thus, β-carotene could be useful for memory enhancement and suggests its potential in the treatment of many neurodegenerative diseases such as Alzheimer’s disease. Although this study did not estimate the effect of β-carotene on tau phosphorylation, which is an important consideration for Alzheimer’s pathogenesis. Future studies are required to be conducted for molecular pathogenesis and treatment of Alzheimer’s. Moreover, the excessive intake of β-carotene is associated with reversible carotenemia.

## Figures and Tables

**Table 1:** The effect of β-carotene following the open field paradigm in mice that received i.c.v STZ.

Parameters			Control	Disease (STZ)	β-Carotene	
Dose mg/kg			CMC 1 mL/kg	3.0	1.02	2.05
Whole body movement	Latency (s.)		2.80 ± 0.374	5.6 ± 0.510	3.20 ± 0.200	2.00 ± 0.316
	Rearing (no.)		27.40 ± 1.030	16.60 ± 0.245	39.60 ± 1.435 **	29.80 ± 9.749
	Freezing (s.)		32.00 ± 1.225 ***	89.00 ± 3.317	29.00 ± 1.789 ***	16.00 ± 2.258 ***
Part body Movement	Scratching (s.)		3.80 ± 0.97 ***	5.40 ± 0.400	2.60 ± 0.400 ***	1.20 ± 0.490
	Teeth Chattering		No	Yes	No	No
	Digging		No	Yes	No	No
Location	Field area Visited	Central (s.)	52.40 ± 7.019 ***	112.00 ± 16.778	30.20 ± 1.985 ***	30.00 ± 3.146 ***
		Peripheral (s.)	209 ± 9.066 ***	88.00 ± 3.742	235.0 ± 2.236 ***	250.080 ± 15.203 ***
	Crossing (no.)		31.6 0 ± 0.927 ***	16.30 ± 0.200	49.40 ± 631 ***	16.00 ± 1.342 ***
ANS	Defecation (no.)		2.60 ± 0.670 ***	17.00 ± 0.447	0.60 ± 0.400 ***	0.400 ± 0.245 ***
	Urination (no.)		4.6 ± 0.400 ***	10.00 ± 0.707	0.80 ± 0.374 ***	0.200 ± 0.200 ***

Data are represented as mean ± SEM, n = 10. (** *P* < 0.01), (**** *P* < 0.001) Significance was given in comparison to the disease group.

**Table 2:** The determination of biochemical markers in brain tissues of mice that received i.c.v STZ.

Sr. #	Treatment Groups	Dose (mg/kg)	GSH (µg/mg of Brain Tissue)	SOD (µg/mg of Brain Tissue)	CAT (µg/mg of Brain Tissue)	Acetylcholine Esterase (µmol/mg of Brain Tissue)
1	Control	CMC (1 mL/kg)	14.4 ± 1.03 ***	10.320 ± 0.185 ***	4.96 ± 0.163 ***	2.8 ± 0.170 ***
2	Diseased (STZ)	300	3.54 ± 0.383	0.043 ± 0.002	1.11 ± 0.206	7.54 ± 0.220 ***
3	Standard (Piracetam)	200	9.40 ± 0.678 ***	0.262 ± 0.020	4.93 ± 1.00 ***	4.620 ± 0.206 ***
4	β-carotene	1.02	10.30 ± 0.200 ***	0.284 ± 0.012	6.15 ± 0.01 ***	3.430 ± 0.187 ***
		2.05	10.320 ± 0.185 ***	0.492 ± 0.012	6.84 ± 0.01 ***	3.380 ± 0.080 ***

Data are represented as mean ± SEM, n = 10. *** *P* < 0.001 Significance was given in comparison to the disease group.

**Table 3:** Binding affinity, inhibition constant, and interactions of the test and standard with AChE.

Compound	Binding Energy (ΔG) kcal/mol	Inhibition Constant (Ki) μM	Interacting Residues	Interaction Type
β-carotene	−7.7	2.27	LEU456, LEU450, LEU127, MET83, VAL71, MET90	Alkyl
Piracetam	−5.8	56.05	TYR130, GLY118, GLU199, SER200, GLY441, TRP84, PHE330	H-Bonding, Alkyl
